# The Relationship Between Authentic Leadership, Psychological Empowerment, Role Clarity, and Work Engagement: Evidence From South Africa

**DOI:** 10.3389/fpsyg.2020.01973

**Published:** 2020-08-18

**Authors:** Tasmin Towsen, Marius Wilhelm Stander, Leoni van der Vaart

**Affiliations:** Optentia Research Focus Area, School of Industrial Psychology and Human Resource Management, North-West University, Vanderbijlpark, South Africa

**Keywords:** authentic leadership, psychological empowerment, role clarity, work engagement, mining organization, South Africa, indirect effects, moderated mediation

## Abstract

Employees in the mining sector are faced with a demanding work environment due to external challenges impacting on the organization. Optimizing their engagement is vital in weathering a demanding environment. The aim of this study was to (a) position authentic leadership (AL) and psychological empowerment (PE) as enablers of work engagement (WE); (b) to investigate the processes (i.e., PE) through which AL exerts its effect on WE, and (c) to determine whether contextual factors [i.e., role clarity (RC)] influenced this process. A cross-sectional research design was employed to collect data from 236 employees employed by a coal-mining organization within South Africa. The AL inventory, PE questionnaire, measures of role conflict and ambiguity questionnaire, and UWES-9 was administered to collect data. A moderated-mediation investigation was employed to test the hypotheses. Results supported the value of AL to enhance WE, both directly and indirectly via PE. Results also concluded that AL exerts its influence on WE through PE, regardless of employees’ levels of RC. AL literature is limited, not only in the South African context but also in the mining sector. The study not only extends AL literature by investigating its outcomes in a South African mining organization, but it also does so by investigating the boundary conditions under which AL exerts its influence. The boundaries (i.e., moderation) within which leadership-subordinate relationships (i.e. mediation) function are often neglected in favor of simplified investigations of mediation processes only.

## Introduction

The South African mining sector is a significant economic player employing almost half a million individuals (Minerals Council South Africa, 2018). Unfortunately, the sector faces various economic and human capital challenges. From an economic perspective, they must weather high input labor costs (Minerals Council South Africa, 2018) limited investment within the economy, and a curb in the utilization of fossil fuels as a result of a technological transition. Last-mentioned creates an additional challenge for coal mining (PricewaterhouseCoopers, 2018; Yelland and Isa, 2019). From a human capital perspective, the sector has an infamous reputation regarding labor unrest. This sector, amongst others, can benefit from not only mitigating labor disputes but also adapting to an exceedingly diverse and augmented workforce, minimizing unproductive workforce practices, enhancing employee retention, satisfaction and engagement, and improving the overall employee experience (Ernst and Young, 2018; Deloitte, 2019).

Given the afore-mentioned human capital challenges, organizations should invest in creating a positive employee experience. Such experiences cultivate work engagement (WE) (Deloitte, 2019). WE is a positive psychological work-related state in which an employee feels energized, committed and deeply involved in tasks (Kahn, 1990; Schaufeli et al., 2002). Employees who experience WE perceive themselves as capable of coping with challenges in their work environment (Bakker et al., 2014; Bakker and Albrecht, 2018). Research on WE proliferated significantly over the past years, indicating the relevance thereof for modern organizations (Bakker and Albrecht, 2018). Hence, organizations are encouraged to identify the determinants of WE.

One such determinant is positive leadership (Lu et al., 2018). Recent research has sparked interest in positive forms of leadership and more specifically authentic leadership (AL) to mitigate organizational challenges (Sidani and Rowe, 2018; Weiss et al., 2018) and to increase positive individual and organizational outcomes, including WE (Bailey et al., 2017). AL is a leader’s ability to put forward an accurate portrayal of themselves, to display an awareness of their behavior and the impact thereof on others, and to demonstrate positive and transparent behavior (Walumbwa et al., 2008; Smith et al., 2019). Authentic leaders can act as custodians of moral aspects within organizations and foster positive self-development among followers (Walumbwa et al., 2008).

Authentic leaders not only directly facilitate WE, but they also indirectly do so by playing an essential role in empowering employees psychologically (Azanza et al., 2018; Du Plessis and Boshoff, 2018b). Psychological empowerment (PE) is an intrinsic motivational construct, based on an employee’s four cognitions of meaning, competence, self-determination, and impact relating to their roles (Spreitzer, 1995; Liu et al., 2019). Employees who perceive their leaders as authentic would experience increased WE in part as a result of them experiencing themselves as psychologically more empowered by their leaders.

Although employees may experience WE as an (indirect) result of AL, the relationship may not be as straightforward as is often assumed. Leadership initiates a motivational process leading to WE (Schaufeli, 2015). However, its association may be impacted on by other contextual factors (Pawar and Eastman, 1997; Oc, 2018). Leaders may become more (or less) effective depending on these contextual factors. One such factor, role clarity (RC), may be relevant. RC is defined as clear and transparent goals and tasks possessed by employees, as well as an understanding of the expected levels of performance and how to achieve such (Rizzo et al., 1970; Flatau-Harrison et al., 2020) and has an inverse relationship with role ambiguity (Rizzo et al., 1970; Hong et al., 2004). Employees who experience more RC are likely to experience heightened levels of WE (De Villiers and Stander, 2011). In line with the job demands-resources (JD-R) model, job resources are particularly necessary when job demands are high, or the context in which the organization finds itself is challenging, such as the mining industry (Bakker et al., 2007). RC is positioned as a job resource to increase WE (Schaufeli, 2012). The relationship between AL and WE may be strengthened when RC is high, rather than low, as RC will act as an additional job resource that amplifies the impact of leadership.

Although AL has been researched extensively, a review of AL studies highlighted several gaps. First, the majority of research is conducted internationally. It is important to note that findings from international studies cannot simply be extrapolated for use within the South African context, as authors argue that leadership is contextually based (Scheepers and Elstob, 2016; Lemoine et al., 2019). Second, AL remains somewhat unexplored in the mining sector as the majority of research is focused on the healthcare sector (Boamah et al., 2016; Coxen et al., 2016; Du Plessis and Boshoff, 2018b) or a combination of healthcare and mining (Du Plessis and Boshoff, 2018a). The challenges faced by leaders (and their subordinates) in the mining industry (i.e., more physical) is vastly different from those experienced in the healthcare industry (i.e., more emotional). Haddon et al. (2015); Malila et al. (2017) advocate for research on the effectiveness of different leadership styles across varying contexts. Third, studies mostly focus on either mediation or moderation. Hence, the processes (i.e., mediation) through which leadership exerts its influences are studied independently from the boundary conditions (i.e., moderation) under which these processes operate. Given that contextual factors may influence leadership effectiveness and processes, a combined (i.e., moderated-mediation) process may further our understanding of leadership.

To address aforementioned theoretical gaps, the current study aimed to investigate the associations between AL, PE, and WE in a coal-mining organization. It also aimed to explore whether AL exerts its influence through PE and whether this indirect effect is enhanced by RC.

## Literature Review

### Authentic Leadership and Psychological Empowerment

Authentic leadership places a focus on those positive aspects which should be augmented and that allow individuals to flourish (Luthans et al., 2001; Avolio and Gardner, 2005; Malila et al., 2017). AL is a leader’s ability to (a) display awareness of themselves and the impact they have on others (i.e., *self-awareness*), (b) act in line with their morals and values and to withstand pressure from others (i.e., *internalized moral perspective*), (c) objectively analyze all available information and views, including those which may differ from their own, to make balanced decisions (i.e., *balanced processing*), and (d) act with transparency in dealings with others (i.e., *relational transparency*) (Walumbwa et al., 2008; Gill et al., 2018). Through exhibiting transparency, authentic leaders are inclined to develop sound, trusting relationships with followers (McAuliffe et al., 2019). The type of relationship which exists between leader and follower is essential to AL, as the authentic leader will be inclined to motivate and inspire followers (Neider and Schriesheim, 2011). Employees would also feel increasingly empowered, in the event where their leaders’ behaviors are viewed as positive (Laschinger et al., 2014) and AL is a form of positive leadership (Laschinger et al., 2014; Zhang et al., 2018).

Spreitzer (1995) developed a validated and empirical multidimensional model of PE, comprising four cognitions, these being meaning, competence, self-determination, and impact. *Meaning* relates to the degree of perceived importance individuals attach to their work or specific tasks (Spreitzer, 1995). Those employees who are empowered are likely to experience augmented meaning from their work (Avolio et al., 2004). *Competence*, or self-efficacy, as a psychological condition of PE can be described as those proficiencies which allow an employee to complete tasks effectively and the perceived degree to which such capabilities exist (Spreitzer, 2008; Chen et al., 2018). *Self-determination* exists when an employee feels motivated to freely, and without coercion, initiate and continue job functions or tasks in an autonomous manner (Spreitzer, 1995; Chen et al., 2018). *Impact* refers to the degree of perceived control of work-related outcomes (Spreitzer, 1995; Mishra and Spreitzer, 1998). Empirical evidence supports the positive relationship between AL and PE (Avolio et al., 2004; Joo and Jo, 2017; Xu and Yang, 2018; Zhang et al., 2018). Based on the discussion above, the following hypothesis was formulated:

Hypothesis 1: Authentic leadership is positively related to PE.

### Authentic Leadership and Work Engagement

Authentic leaders not only empower their followers, they also enhance their engagement in their work. WE is a work-related psychological state of mind, positive in nature and an indicator of employee well-being (Schaufeli et al., 2002). WE consists of three dimensions, namely vigor, dedication, and absorption (Schaufeli et al., 2002). Vigor is operationalized as the degree of effort and energy an employee is willing to put into his or her work (Ahmad and Gao, 2018). Vigor enables an employee to apply mental resilience to counteract challenges within the work environment (Bakker and Albrecht, 2018). Dedication is operationalized as the amount of importance an employee attaches to his or her work (Ahmad and Gao, 2018). When referring to dedication in terms of WE, employees experience a degree of desirable challenge in their work (De Beer et al., 2016). Absorption is described and operationalized as being fully immersed in or concentrated on one’s work, to such a degree that an individual may enter a state of “flow” (Schaufeli et al., 2002; Schaufeli and Bakker, 2004; Ahmad and Gao, 2018). Authentic leaders influence their followers’ work attitudes (i.e., engagement) by means of identification and through eliciting hope, trust, and positive emotions. In other words, subordinates become more engaged in their work because they identify with the leader and the collective (i.e., team), they are more hopeful, they trust their leader, and they experience more positive emotions (Avolio et al., 2004). Empirical evidence supports the positive relationship between AL and WE (Hsieh and Wang, 2015; Joo et al., 2016a; Scheepers and Elstob, 2016; Oh et al., 2018; Álvarez et al., 2019). Based on the discussion above, the following hypothesis was formulated:

Hypothesis 2: Authentic leadership is positively related to WE.

### Psychological Empowerment and Work Engagement

Like AL, PE also enhances WE. Positive organizational outcomes can result from PE, as employees’ experience enhanced perceived control over work factors and intrinsic motivation to engage in work tasks (Quinn and Spreitzer, 1997). When engaging in their work, employees may evaluate how meaningful their behaviors are, how competent they are, how much autonomy they have in performing their tasks, and how much of an impact their behaviors will have. Given that empowered employees believe that they perform meaningful and impactful behaviors and that they believe in themselves and experience a sense of freedom in their tasks, they are more engaged (Stander and Rothmann, 2010). Empirical evidence supports the theorized positive relationship between PE and WE (Stander and Rothmann, 2010; Bhatnagar, 2012; Jose and Mampilly, 2015). Based on the discussion above, the following hypothesis was formulated:

Hypothesis 3: Psychological empowerment is positively related to WE.

### Authentic Leadership, Psychological Empowerment, and the Moderating Role of Role Clarity

The relationship between AL and PE may be influenced by RC. RC refers to the degree of clarity and certainty surrounding task and performance expectations and the extent to which information relating to performance expectations is made available, as well as suitable behaviors associated herewith (Frogeli et al., 2019). RC is more than merely an employee’s job description and relates to the clearly defined and transparent expectations from both the employee and the leader acting on behalf of the organization – transparent expectation management (Yadav and Rangnekar, 2016). Caker and Siverbo (2018) define RC as the extent to which employees are aware of what is expected to be achieved, and how to go about achieving such. Role ambiguity and role conflict have the likelihood to prevail in the absence of clearly defined job roles since an incongruity between actual tasks performed and expected tasks to be performed will exist (Yadav and Rangnekar, 2016). RC, positioned as a job resource, is postulated to lead to increased levels of PE (Bakker and Demerouti, 2017) and the absence hereof may lead to demising positive psychological states, such as PE, due to employees having to employ coping mechanisms (Lau, 2015).

Organizational leaders play an imperative role in providing and ensuring RC through sufficient levels of information and ensuring that information is made available to employees relating to expected roles and tasks, and associated performance levels are clear (Whitaker et al., 2007). A transparent communication style is necessary on the part of the leader to ensure RC (Yadav and Rangnekar, 2016). Should a leader not effectively provide RC, a resource, the impact of a leader on an employee’s PE will likely be weakened, as employees respond to supportive leadership which provides resources. One such resource may be RC and it will positively impact upon an employee’s PE (Nel et al., 2015; Vullinghs et al., 2018). Empirical evidence supports the positive relationship between PE and RC (Baron and Kenny, 1986; Spreitzer, 1995) but also the moderating effect of RC between leadership behaviors and employee outcomes (Orgambídez and Almeida, 2020). Based on the discussion above, the following hypothesis was formulated:

Hypothesis 4: Role clarity will moderate the relationship between AL and PE, such that the relationship between AL and PE will be stronger for individuals with high rather than low RC.

### The Indirect Effect of Authentic Leadership and the Boundary Conditions of Role Clarity

The effect of AL on WE may be a complex relationship. Studies do not always demonstrate significant relationships between AL and its theoretically proposed outcomes. In the South African public healthcare sector, studies indicated that AL neither had a significant influence on organizational citizenship behavior (Coxen et al., 2016) nor with WE (Stander et al., 2015). These findings could indicate that AL may not always have its intended consequences or that other variables explain (i.e., mediate). For example, leaders are essential in psychologically empowering employees, and by empowering subordinates, it indirectly enables WE (Azanza et al., 2018). AL, a positive form of leadership, can contribute to employees’ perceived experience of PE (Xu and Yang, 2018). Employees who experience PE feel they have more control over their work tasks, experience intrinsic motivation, and can better adapt to demands, which in turn leads to increased WE (Quinn and Spreitzer, 1997; Fisher, 2014). Previous studies support the mediating role of PE. For example, PE mediated the association between ethical leadership and WE (Ahmad and Gao, 2018) between AL and employee creativity (Mubarak and Noor, 2018) between leader-member exchange (LMX) and employee voice (Wang et al., 2016) and between empowering leadership behavior and WE (Rayan et al., 2018).

Hypothesis 5: Authentic leadership has an indirect effect on WE through PE.

Leadership effectiveness and the processes through which leaders influence followers may also be influenced by contextual factors (i.e., moderators). One such contextual factor, RC, may be relevant. RC is the degree to which an employee feels enlightened, relating to what is expected of them and how this is expected to be achieved (Frogeli et al., 2019). Employees who lack RC will be less inclined to experience PE (Hall, 2008) resulting in negative consequences for WE. In a study conducted by Wang et al. (2016) RC moderated the relationship between LMX and PE in such a manner that increased levels of RC strengthened the relationship between LMX and PE. If (a) both AL and RC hold the potential of enhancing PE, (b) AL indirectly influences WE through PE, and (c) RC acts as a moderator in the leadership processes, then it can be postulated that RC may strengthen the (indirect) effect of AL.

A moderated-mediation model is proposed in which authentic leaders create more psychologically empowered employees and, in turn, more engaged employees. These leaders are more successful in doing so when employees are also clear about the roles and performance expectations. Based on the discussion above, the following hypothesis was formulated:

Hypothesis 6: Role clarity will moderate the effect of AL on WE via PE, such that the indirect effect will be stronger for individuals with high rather than low RC.

## Materials and Methods

### Approach

A quantitative approach was utilized in the current study. This approach involves collecting numerical data relating to the research participants in order to establish a method in which inferences can be made via statistical analysis (Kumar, 2019). A cross-sectional survey design was used, involving taking a snapshot of a population group through data collection at one specific time (Kumar, 2019). Cross-sectional survey designs allow for an overview of a phenomenon at a specific point in time (Kumar, 2019) and were therefore suitable for this study. Secondary data was utilized during the research study, but primary data analysis was performed.

### Participants

The research participants (*n* = 236) from whom the data had been obtained were employees of a coal-mining organization based in South Africa. A letter requesting participation, together with a consent form, was distributed in envelopes to all 300 staff members on Paterson B level and above. This was followed up with two reminders. The majority of the participants were African (55.08%) males (87.29%). Concerning the age groups, the 26–35 age group was the most representative category (32.20%) followed by the 36–45 years’ age group (30.08%). Almost half of the participants were working in the engineering department (49.58%), followed by the operations (23.73%), technical (8.47%), human resources (5.51%), and finance (1.69%) departments. Of the participants, the majority (75.00%) were employed at the C1–C4 level. In terms of tenure, the 6–10 years’ category was the most representative (28.39%), closely followed by the 1–5 years’ experience category (27.12%).

### Measures

#### Measurement Instruments Utilized in the Study

##### Biographical questionnaire

A biographical questionnaire was utilized to determine the information relating to the biographical features of the research participants.

##### Authentic leadership

For the purpose of measuring the construct of AL – the AL inventory (ALI) developed by Neider and Schriesheim (2011) was utilized. The ALI was adapted from the original work of Walumbwa et al. (2008). The measuring instrument contained 16 items and was scored on a five-point Likert type scale, ranging from 1 being “disagree strongly” to 5 being “agree strongly.” The ALI measured the four dimensions of AL, these being: self-awareness (4 items); balanced processing (4 items); internalized moral perspective (4 items); and relational transparency (4 items). Example items from the ALI include “My leader shows that he/she understands his/her strengths and weaknesses” (self-awareness), “My leader carefully listens to alternative perspectives before reaching a conclusion” (balanced processing), “My leader uses his/her core beliefs to make decisions” (internalized moral perspective), and “My leader openly shares information with others” (relational transparency).

##### Psychological empowerment

For the purpose of measuring the construct of PE – the PE questionnaire (PEQ) developed by Spreitzer (1995) was utilized. The PEQ contained 12 items and was scored on a seven-point Likert type scale, 1 being “very strongly disagree” and 7 being “very strongly agree.” The PEQ measured the four dimensions of PE, being: meaning (3 items), competence (3 items), self-determination (3 items); and impact (3 items). Example items from the PEQ include “The work I do is meaningful to me” (meaning), “I have mastered the skills necessary for my job” (competence), “I have significant autonomy in determining how to do my job” (self-determination), and “I have significant influence over what happens in my department” (impact) (Spreitzer, 1995).

##### Role clarity

For the purpose of measuring the construct of RC, the measures of role conflict and ambiguity questionnaire (MRCAQ) developed by Rizzo et al. (1970) was utilized. Four items from the MRCAQ were employed to measure RC during data collection and the items were scored on a five-point Likert type scale, 1 being “strongly disagree” and 5 being “strongly agree.” Example items from the MRCAQ include “I know what my responsibilities are” and “I know exactly what is expected of me.”

##### Work engagement

For the purpose of measuring the construct of WE – the UWES-9 (Schaufeli et al., 2006) which is the shortened version of the 17-item Utrecht WE Scale (Schaufeli and Bakker, 2004) was used. The measuring instrument included nine items and was scored on a six-point Likert type scale, ranging from 1 being “never” to 6 being “always.” The UWES scale measured the three dimensions of WE: vigor (3 items), dedication (3 items), and absorption (3 items). Example items included were: “At my job, I feel strong and vigorous” (vigor), “I find the work I do full of meaning and purpose” (dedication), and “When I am working, I forget everything else around me” (absorption).

### Research Procedure

The data was collected as part of a larger research project focusing on a coal mine in South Africa. The objectives of the research had been explained via the consent form. Site visits, along with Human Resources personnel, occurred to answer any queries that the participants may have had, as well as to explain the purpose and confidentiality of the study. All participants expressed their willingness to participate in the study by completing a consent form.

### Statistical Analysis

**FIGURE 1:**
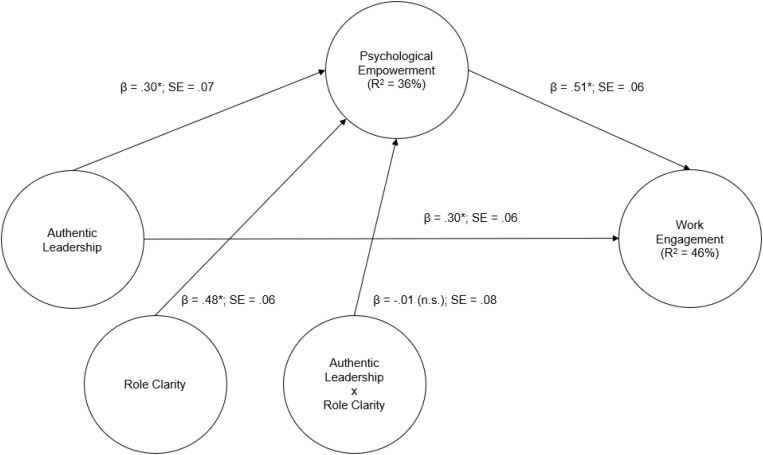
Results for the hypothesized moderated-mediation model.

**FIGURE 2:**
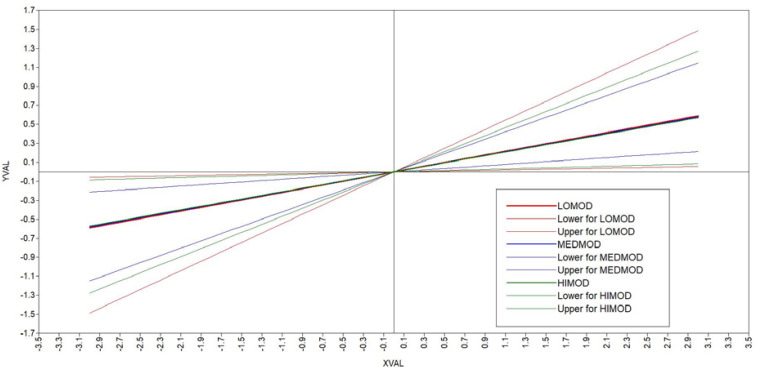
Conditional indirect effects represented graphically with 95% confidence interval.

Mplus 8.3 was used to perform the analyses for this moderated-mediation investigation (Muthén and Muthén, 1998). A latent variable approach was followed within a confirmatory factor analytic (CFA) framework with maximum likelihood estimation (Brown, 2015). Specifically, two types of models were tested: The first, a measurement model that included all of the study variables in one model as second-order (higher-order) factors indicated by the corresponding first-order factors [e.g., PE (second-order factor) indicated by its four first-order sub-component factors (meaning, competence, impact, and self-determination – which were in turn estimated with their corresponding items)]. This second-order model was used as the basis for investigating the validity of the measurement model and for making any modifications as indicated by the results. The second model was the (structural) moderated-mediation model (Figure 1). For this model, the necessary input coding was substituted in a template available online that allows for the testing of moderated-mediation with latent variables in Mplus in line with Hayes (2013) model 7 (Stride et al., 2015). To ascertain the fit of the models, the following fit indices were considered: CFI ≥ 0.90, TLI ≥ 0.90, and RMSEA < 0.08 (Van de Schoot et al., 2012). Additionally, for the structural model, 5 000 bootstrap replications were specified as part of the input for the estimation process of the parameters. The bootstrap replications allow for a more accurate estimate of the indirect effect(s) as it generates 95% confidence intervals that should not cross zero (Hayes, 2017). A plot was also generated to display the potential moderated-mediation effect at the lower, middle, and upper range of its estimates (Figure 2).

For the descriptive statistics, RStudio version 1.1.463 (R Studio Team, 2016) was used with R base-version 3.5.2 (R Core Team, 2018). In RStudio, the *psych* packages *describe* function was used to calculate the means and standard deviations for each of the factors in the model (Revelle, 2018). To calculate the composite reliability coefficients of the factors, the *scale reliability* function was used from the *userfriendlyscience* package (Peters, 2018) these values were added to the correlation table generated from the latent variable estimation in Mplus (Table 1). For reliability of the scales the general rule of thumb of 0.70 and above was considered, and for the correlations, the standard cut-off criteria were used for effect sizes: *r* = 0.10–0.29 (small effect), *r* = 0.30–0.49 (medium effect), and *r* ≥ 0.50 (large effect) (Cohen, 1992).

**TABLE 1:** Reliabilities, descriptive, and correlation statistics for the variables (*n* = 236).

Variables	ρ	Range	M	SD	1	2	3	4	5	6	7	8	9	10
1. Authentic leadership	0.95	1–5	3.47	0.78	1.00									
2. Vigor	0.80	1–6	4.16	1.28	0.48*	1.00								
3. Dedication	0.87	1–6	4.73	1.28	0.48*	0.98**	1.00							
4. Absorption	0.79	1–6	4.41	1.21	0.45*	0.92**	0.92**	1.00						
5. Work engagement	0.92	1–6	4.43	1.13	0.49*	0.99**	0.99**	0.93**	1.00					
6. Role clarity	0.88	1–5	4.54	0.65	0.15	0.37*	0.37*	0.35*	0.37*	1.00				
7. Meaning	0.91	1–7	5.78	1.15	0.25	0.40*	0.40*	0.38*	0.41*	0.34*	1.00			
8. Competence	0.89	1–7	5.93	0.89	0.27	0.44*	0.44*	0.41*	0.44*	0.37*	0.48*	1.00		
9. Self-Determination	0.82	1–7	5.44	1.11	0.32*	0.53**	0.53**	0.50**	0.53**	0.44*	0.58**	0.63**	1.00	
10. Impact	0.86	1–7	4.99	1.30	0.31*	0.51**	0.51**	0.48*	0.52**	0.43*	0.56**	0.61**	0.73**	1.00
11. Psychological empowerment	0.92	1–7	5.54	1.11	0.37*	0.61**	0.61**	0.57**	0.61**	0.51**	0.66**	0.72**	0.87**	0.84**

ρ = composite reliability coefficient; M = mean; SD = standard deviation; All correlations statistically significant *p* < 0.05; * = Medium effect; ** = Large effect.

## Results

### CFA: Fitting the Measurement Model

The second-order model, as described above, was specified and estimated with maximum likelihood. However, the results indicated that specifying AL as a second-order factor based on its first-order factors led to a non-positive definite matrix. A variant was then estimated where AL was considered by its first-order factors only, removing the second-order constraint for that specific factor. But this model also presented validity challenges with correlations above 0.90 and even above 1.00. The problem was solved by specifying AL as a single first-order factor indicated by all of the items as was also done in the study by Stander et al. (2015). This model presented the following fit statistics: χ^2^ = 1543.31, *df* = 727, CFI = 0.88, TLI = 0.87, and RMSEA of 0.07 – missing the rule of thumb thresholds. To address this possible concern, modification indices were inspected and six modification constraints were added to the model, all of them correlations between the error residual variances between specific items: (1) AL1 [“My leader solicits (asks) feedback for improving his/her dealings with others”] with AL4 (“My leader asks for ideas that challenge his/her core beliefs”); (2) AL13 (“My leader is clearly aware of the impact he/she has on others”) with AL14 (“My leader expresses his/her ideas and thoughts clearly to others”); (3) EMP11 (“I have a great deal of control over what happens in my department”) with EMP12 (“I have significant influence over what happens in my department”); (4) ENG1 (“At my work, I feel bursting with energy”) with ENG2 (“At my job, I feel strong and vigorous”); (5) ENG3 (“When I get up in the morning, I feel like going to work”) with ENG9 (“I get carried away when I’m working”), and (6) ENG7 (“I am proud of the work that I do”) with ENG8 (“I am immersed in my work”). This final re-specified model indicated the following fit statistics: χ^2^ = 1374.18, *df* = 721, CFI = 0.90, TLI = 0.90, and RMSEA of 0.06. Therefore, this model constituted the final measurement model which served as the foundation for the (structural) moderated-mediation model.

### Reliabilities, Descriptive Statistics, and Correlation Matrix

Table 1 below presents the reliabilities for each of the factors, means, standard deviations, and correlations for the variables.

As can be seen, all of the variables had acceptable reliabilities (ρ > 0.70). The means and the standard deviations were all within the acceptable range. In terms of the correlations, all of the relationships were statistically significant (*p* < 0.05). Specifically, all of the first-order factors had high correlational values with the corresponding second-order latent variable – indicating validity for the second-order approach taken. For example, WE was highly correlated with vigor (*r* = 0.99), dedication (*r* = 0.99), and absorption (*r* = 0.93) – all large effects. Given the significance of all the correlations, which provided preliminary evidence for some of the hypotheses: AL correlated with PE (*r* = 0.37; medium effect) and WE (*r* = 0.49; medium effect/borderline large effect). PE had a positive correlation with WE (*r* = 0.61; large effect). Furthermore, the moderator – RC – had positive correlations with AL (*r* = 0.15; small effect), PE (*r* = 0.51; large effect), and WE (*r* = 0.37; medium effect).

### Structural Model: Direct Paths and Conditional Indirect Relationships

Due to the numerical integration used in the estimation process of moderated-mediation models in Mplus with latent variables, no traditional fit statistics are provided for the model. However, the model is based on the final measurement model of which fit statistics are provided above.

The direct path results showed that AL had a significant relationship with PE (β = 0.30, SE = 0.07; supporting H1) and WE (β = 0.30, SE = 0.06; supporting H2). Furthermore, Table 2 shows PE had a significant relationship with WE (β = 0.51, SE = 0.06; supporting H3). As part of the statistical model, RC had a significant relationship with PE (β = 0.48, SE = 0.06). Lastly, the interaction effect was non-significant – indicating no moderating effect (β = −0.01, SE = 0.08; rejecting H4). Figure 1 below graphically depicts the results from the statistical model.

**TABLE 2:** Path results for the structural statistical moderated-mediation model.

Structural path	β	SE	*p*	Result
Authentic leadership → Psychological empowerment	0.30*	0.07	0.001	Significant
Authentic leadership → Work engagement	0.30*	0.06	0.001	Significant
Psychological empowerment → Work engagement	0.51*	0.06	0.001	Significant
Role clarity → Psychological empowerment	0.48*	0.06	0.001	Significant
Interaction effect → Psychological empowerment	–0.01	0.08	0.956	Not significant

β = standardized beta coefficient; SE = standard error; *p* = two-tailed statistical significance; * = *p* < 0.001.

The indirect effect from AL to WE through PE was found to be a significant effect as the 95% confidence interval for the estimate did not include zero (Estimate = 0.193; 95% CI [0.071, 0.382]; H5 supported). Table 3 below presents the conditional indirect effects with each condition of RC as moderator (low, medium, high) with corresponding 95% confidence intervals.

**TABLE 3:** Conditional indirect relationships for the moderated-mediation model.

Conditional indirect effects	Estimate	95% CI	95% CI
		Lower Limit	Upper Limit
Index of moderated-mediation (IMM)	–0.003	–0.162	0.147
Condition: Low role clarity	0.567*	0.278	0.917
Condition: Medium role clarity	0.564*	0.342	0.860
Condition: High role clarity	0.561*	0.313	0.862

Unstandardised estimates as standardized are not available with bootstrapping and numerical integration. * = does not include zero.

As can be seen the IMM included zero indicating the untrustworthiness of the parameter (Estimate = −0.003; 95% CI[−0.162, 0.147] – rejecting H6), and for the conditional indirect effects for each of the levels of the moderator (RC) the values are the same and not significantly different from another – supporting the lack of moderation by RC under low, medium or high conditions of the variable. Figure 2 below presents the graph of the non-moderation for each condition of the variable with 95% confidence intervals and according to the analysis these values did not significantly differ.

## Discussion

The aim of this study was to position AL (as a job resource) and PE (as a personal resource) as enablers of WE; to investigate the processes (i.e., PE) through which AL exerts its effect on WE and to determine whether contextual factors, in the form of RC, influenced the relationship between AL, PE, and WE. Engaged employees are key in the South African mining sector due to the challenges and demands experienced in this sector (McKinsey Company, 2019). WE ranges among the most important organizational metrics for people efficiency (Bakker et al., 2014; Bakker and Albrecht, 2018), and is widely established in both the academic and professional literature as a driver of positive outcomes (Roberts and Davenport, 2002; Halbesleben, 2010; Olugbade and Karatepe, 2018).

Results indicated a positive relationship between AL and PE and AL and WE – higher levels of AL lead to greater levels of PE and WE. This evidence supports postulating AL as a job resource consistent with the JD-R model (Bakker and Demerouti, 2017; Lee et al., 2020) and is in line with previous empirical studies (Joo and Jo, 2017; Wang et al., 2017; Du Plessis and Boshoff, 2018b; Zhang et al., 2018; Adil and Kamal, 2019; Álvarez et al., 2019). The results mean that being aware of yourself as a leader and the impact you have on others, together with being transparent and including others’ opinions, creates confident employees who feel they are self-determined and can make a difference in their work while deriving meaning from it. One can expect that the leader with a high degree of self-awareness and openness will create feelings of trust in direct reports. Experiencing trust from their leaders will contribute to trust in employee’s own abilities as well as their wellness. AL is also a valuable resource in creating dedicated, energized subordinates that are absorbed in their work. AL is a form of positive leadership (Laschinger et al., 2014; Zhang et al., 2018) that will build trusting relationships (McAuliffe et al., 2019) leading to motivated followers (Neider and Schriesheim, 2011).

A relationship was also established between PE and WE. This evidence supports postulating PE as a personal resource consistent with the JD-R model (Bakker and Demerouti, 2017, 2018) and is in line with various studies in this domain (Stander and Rothmann, 2010; Jose and Mampilly, 2015). Psychologically empowered employees will experience heightened levels of autonomy, control over their work, perceived competence, and an enhanced sphere of influence (Wang et al., 2016). The results mean that employees, who experience higher levels of meaning and attachment to their roles, are self-determined, feel confident in the work they are performing and perceive their contributions to be impactful will be increasingly engaged in their jobs. The mining industry can be described as a high-risk and harsh work environment. In a challenging work environment, PE (as a personal resource), plays an essential role in the way that employees apply their energy to counter challenges in the work environment. Given that empowered employees believe that they perform meaningful and impactful behaviors and that they believe in themselves and experience a sense of freedom in their tasks, they are likely to be more engaged (Stander and Rothmann, 2010).

Support was established for an indirect relationship between AL and WE through PE. Leadership, as a resource, initiates a motivational process (Schaufeli, 2015; Schaufeli et al., 2019). This motivational process will lead to employees experiencing heightened levels of PE (Quinones et al., 2013) and, in turn, lead to heightened levels of WE. AL allows for positive self-development and autonomy on the part of followers, input from employees, the encouragement of open sharing of information and feedback relating to personal efficacy, hereby establishing higher-quality relationships which foster empowerment and diminish feelings of powerlessness on the part of the employee (Wong and Laschinger, 2012). Employees who experience PE feel they have more control over their work tasks, experience intrinsic motivation, and can better adapt to demands, which in turn leads to increased WE (Fisher, 2014; Quinn and Spreitzer, 1997). Leaders enhancing employees’ levels of PE and WE will contribute to creating a positive employee experience.

Contrary to theoretical expectations, no support was found for the hypothesis that the direct and indirect associations between AL and PE will be moderated through RC. In essence, having higher or lower levels of RC will not influence the size or the direction of the relationship between AL and PE. Contrary to expectations, AL will indirectly influence WE regardless of the levels of RC. Early possibilities may be the close nomological proximity of AL and RC; both of which require a mature organizational culture, a strong sense of transparency and clarity on expectations. It is possible to argue that, when AL exists in an organization, the employees of such organization should inherently be clearer on what is expected of them, the core outcomes of their roles and what they need to do in order to advance organizational goals. Authentic leaders openly disclose information and hold no hidden intentions or purposes (Wong and Cummings, 2009). One can assume that transparency is not limited to the task. This corresponds with Yadav and Rangnekar (2016) opinion that RC is more than only clarity on employee’s job description and relates to defined and transparent role expectations, performance expectations as well as suitable behaviors (Frogeli et al., 2019). Another possible explanation for the non-significant influence of RC may be that it differs between leaders, departments, or functions. Some roles and functions are more standardized with less room for self-interpretation. It is, however, important to note that the correlation between AL and RC was not significant; therefore, it may be more plausible that the findings illustrate and emphasize how powerful the effect of AL is on employee outcomes.

This research addressed the theoretical gaps mentioned. The positive influence of AL on PE and WE illustrates the value of AL, not only broadly in the South African context but also, more specifically in the mining industry. In this way, the findings of the current study may also extend to other countries that rely on the mining industry for economic growth or perhaps other industries or organizations that are equally physically challenging. Although an understanding of boundary conditions of AL’s mechanisms is important for theory development and practice, mediated-moderation studies in the domain of AL are limited. The current study aimed to contribute in this way, by shedding some light on the boundary conditions that may be eliminated in the quest for a better understanding of the leadership-subordinate influencing process.

### Managerial Implications

The findings of this study provide two avenues for organizations to develop more engaged employees: through developing authentic leaders and enhancing PE. The first avenue proposes that organizations introduce interventions to enhance AL. This can be done through ensuring that a mature organizational culture is in place which encourages authenticity and leaders to undertake a self-awareness journey and clearly define and understand their unique leadership value proposition in terms of values and motivations.

The second avenue advocates that organizations introduce interventions allowing employees the freedom to pursue inherently rewarding, meaningful and impactful job roles and hereby enhance PE, leading to increased levels of WE. The four cognitions of PE are to be enhanced in order to enhance overall perceptions of PE. This can be achieved through organizations allowing for job crafting as a personal strategy to take place, whereby employees are able to autonomously craft their jobs to create growth opportunities and enhanced meaning (Kohll, 2018).

### Limitations of the Study and Recommendations for Future Studies

When interpreting the results of the current study, several methodological limitations should be taken into account. First, the cross-sectional nature of the study does not allow for casual inferences to be made regarding the variables. Second, common method variance (CMV) may result from a cross-sectional research design, as bias and over-inflation of relationships may be introduced through self-report measures (Podsakoff et al., 2003). Longitudinal research designs may buffer the negative implications of CMV resulting from cross-sectional research designs, as well as design strategies such as measuring variables at differing times and balancing negative and positive items (Podsakoff et al., 2003). However, an inherent assumption in cross-sectional (and even traditional longitudinal) studies is that the constructs under investigation are relatively stable over time (Ohly et al., 2010). We know that each of the variables in the current study fluctuate over time (Zimmerman, 1990; Bakker, 2014; Breevaart et al., 2016; Joo et al., 2016b; Breevaart and Bakker, 2018; Vullinghs and Dóci, 2020). Some days we experience our leaders as more authentic, and we feel more psychologically empowered than other days. Similarly, some days we experience more RC, and we feel more engaged than on others. In order to capture these fluctuations and draw more accurate conclusions about “how” things happen (i.e., relationships) and “why” these things happen (i.e., determinants) (Roe, 2008; Griep and Hansen, 2020) more intensive, dynamic research designs are necessary. For this reason, diary studies are recommended for future research rather than ordinary longitudinal studies. Second, and related to the previous recommendation of differentiating between the within- and between-person levels, the current study did not measure (or matched) leaders or work groups (with followers). Consequently, multi-level analysis could not be performed. As AL may operate on different levels of analysis (i.e., individual, dyad, group, and organization) (Yammarino et al., 2008) it is recommended that researchers measures the variables in a way that will enable a more nuanced understanding of how leadership and its outcomes unfold within and across these four levels (Batistič et al., 2017). Last, generalization to other contexts may be limited due to the fact that the data had been collected from a single mining operation. Future research could expand its reach to include multiple organizations and different industries.

The first theoretical limitation to consider would be that only a limited number of variables had been introduced as resources within the JD-R framework. Future research could focus on exploring how different categories, for example job, personal and even home resources, influence the processes through which AL influences follower outcomes. Examples of moderators could include quality of the leader-subordinate relationship, leaders’ psychological capital, and psychological safety. The second theoretical limitation to consider would be that resources, rather than demands, had been the focal point of the current research. The interaction effect of resources and demands could be explored further. Including more resources and/or demands can be approached from a multi-level perspective to determine whether team and organizational factors act as buffer or exacerbator on individual level outcomes in line with the recommendations by Bakker and Demerouti (2018). In addition to the inclusion of different resources, it is also recommended that future research explore the determinants of AL as this presents as a significant gap in research (Alilyyani et al., 2018) and the current study illustrates the value-add of AL in mining organizations.

## Conclusion

The study illustrated the value of AL (as job resource) to enhance WE, both directly and indirectly by enhancing feelings of PE (a personal resource) among mining employees. Results also concluded that AL exerts its influence on WE through PE, regardless of employees’ levels of RC.

## Data Availability Statement

The raw data supporting the conclusions of this article will be made available by the authors, without undue reservation.

## Ethics Statement

The studies involving human participants were reviewed and approved by Economic and Management Sciences Research Ethics Committee (EMS-REC) (NWU-0092-19A4), Faculty of Economic and Management Sciences, North-West University, South Africa.

## Author Contributions

TT acted as the primary researcher as this study formed part of her master’s research. She shared in the conceptualization of the manuscript, interpretation of the research results, and the writing of the manuscript. MS and LV acted as supervisor and co-supervisor, respectively. They played an advisory role, assisting in the conceptualization of the study, collecting data, assisting with the interpretation of the research results, and refining the research manuscript. Prof. L. T. de Beer analyzed the data. All authors contributed to the article and approved the submitted version.

## Conflict of Interest

The authors declare that the research was conducted in the absence of any commercial or financial relationships that could be construed as a potential conflict of interest.
